# The efficacy of intravenous iron for treatment of anemia before cardiac surgery: An updated systematic review and meta-analysis with trial sequential analysis

**DOI:** 10.1186/s13019-023-02119-2

**Published:** 2023-01-11

**Authors:** Hong-Mei Liu, Xi-sha Tang, Hong Yu, Hai Yu

**Affiliations:** Department of Anesthesiology, West China Hospital, Sichuan University, Chengdu, 610041 China

**Keywords:** Anemia, Intravenous iron, Transfusion, Cardiac surgery, Meta-analysis

## Abstract

**Background:**

Preoperative anemia is common in patients undergoing cardiac surgery with various etiologies, among which iron deficiency is the leading cause. However, the benefit of intravenous (IV) iron for the treatment of anemia before cardiac surgery is uncertain. This updated meta-analysis aimed to evaluate the efficacy of IV iron in adult cardiac surgery patients with preoperative anemia.

**Methods:**

This review was conducted according to Preferred Reporting Items for Systematic Reviews and Meta-Analyses (PRISMA) guidelines. We searched Embase, PubMed and the Cochrane Central Register of Controlled Trials to identify eligible randomized controlled trials (RCTs) and observational studies. Quality was assessed using the Cochrane Collaboration risk of bias tool and Newcastle–Ottawa scale, and the strength of evidence was assessed using the Grading of Recommendations, Assessment, Development and Evaluations (GRADE) criteria. Trial sequential analysis was performed on the primary outcome (transfusion rate) to confirm whether firm evidence was reached.

**Results:**

Six RCTs (936 patients) and 5 observational studies (1350 patients) were included in this meta-analysis. The IV iron group and the control group were comparable in terms of transfusion rate [55.1% vs 60.9%, risk ratio (RR) = 0.91, 95% confidence interval (CI) 0.81–1.03, P = 0.13, low quality]. There were no significant differences in units transfused per patient, ICU stay and hospital length of stay between the two groups. And pooled data showed a benefit of IV iron compared to the control group on mortality (2.76% vs 3.75%, RR = 0.58, 95% CI 0.36–0.95, P = 0.03, moderate quality) and no mortality reduction existed when including only RCTs.

**Conclusions:**

This meta-analysis suggested that IV iron treatment for patients with anemia before cardiac surgery did not reduce the transfusion requirement (low quality), but it was associated with decreased mortality (moderate quality). More large-scale, high-quality randomized clinical trials are warranted to confirm or refute our findings.

*PROSPERO registry reference*: CRD42022331875.

**Supplementary Information:**

The online version contains supplementary material available at 10.1186/s13019-023-02119-2.

## Background

Anemia is a common condition observed in patients scheduled for cardiac surgery with its incidence ranging 20–50% in different circumstances [1, 2]. Anemia remains an independent risk factor for perioperative allogeneic blood transfusion (ABT), associated with increased hospital length of stay (LOS), postoperative morbidity and mortality [3–7]. Because iron deficiency is the most common cause of anemia [8, 9], intravenous (IV) iron therapy has been proposed as an intervention to correct preoperative anemia and reduce ABT perioperatively [9–12].

However, the evidence to support the benefit of preoperative IV iron for the treatment of anemia before cardiac surgery remains highly uncertain. A 2020 meta-analysis [13] involving cardiac surgery patients from 4 randomized controlled trials (RCTs) and 7 observational studies found that IV iron was associated with improved clinical outcomes, including reduced transfusion rates. Nonetheless, another systematic review [14] suggested that the current literature was unable to demonstrate the merits of preoperative IV iron in cardiac surgery. Thus, the request for strong evidence is ongoing in this area of research. In addition, two RCTs and four observational studies focusing on this topic have been published between 2020 and 2022 [3, 15–19].

Based on the emerging data, an updated systematic review and meta-analysis of RCTs and observational studies exploring the efficacy of preoperative IV iron (alone or combined with other agents) for anemic patients undergoing cardiac surgery was conducted. Our primary aim was to evaluate the efficacy of preoperative IV iron therapy in reducing erythrocyte transfusions intra- and postoperatively.

## Methods

### Systematic search

Based on the Preferred Reporting Items for Systematic Reviews and Meta-Analyses (PRISMA) Guidelines [20] (Additional file 1: A) and the recommendations from the Cochrane Collaboration, a systematic search was performed. The protocol for this systematic review and meta-analysis was registered on PROSPERO (CRD42022331875) on May 5th, 2022. The search strategy, including the following keywords: anemia, intravenous, iron and cardiac surgery was performed in PubMed, Embase and Cochrane Central Register of Controlled Trials (CENTRAL) (detailed search strategy in Additional file 1: B). The retrieval time was from the inception of the database to May 3rd, 2022. Ethical approval and patient consent were not required in a meta-analysis.

### Eligibility criteria

Two reviewers (HML and XST) independently assessed all citations to screen eligible articles for a second-stage full-text review. Then full texts were reviewed for eligibility. If there were disagreements, two other reviewers, Y.H1 (Yu Hai) and Y.H2 (Yu Hong), would be consulted. Studies were included if they met the following criteria:

1. Population: adults (> 18 years of age) with preoperative anemia undergoing cardiac surgery.
2. Intervention: IV iron [alone or combined with other agents, such as erythropoietin (EPO) or vitamins]. We limited the complementary treatments to the application of EPO.
3. Comparison: placebo or no treatment or oral iron or EPO or a combination of several of the above four therapies.
4. Outcomes: The primary outcome was transfusion rates (the number of patients who received erythrocyte transfusions intra- and postoperatively). Secondary outcomes included units of erythrocytes transfused per patient, all-cause mortality, intensive care unit (ICU) and hospital LOS, and adverse events [including renal adverse events (acute kidney injury or renal replacement therapy), cardiac adverse events (myocardial injury) and cerebral adverse events (stroke)]. The studies included in this analysis reported at least one of the above outcomes.
5. Design: RCTs and observational studies.

### Data extraction

Two reviewers (HML and XST) independently extracted information. The extracted information included the name of the first author, year of publication, surgery type, sample size and group assignment, iron dose used with or without other agents, time interval between iron therapy and surgery, and outcomes (transfusion rates, units of erythrocytes transfused per patient, all-cause mortality, ICU stay, hospital LOS and adverse events).

### Quality assessment

Two authors independently used the Cochrane Collaboration's risk of bias assessment tool to assess the quality of RCTs from the following seven aspects: random sequence generation, allocation concealment, blinding of participants, blinding of outcome assessment, incomplete outcome data, selective outcome reporting, and other sources of bias. The included RCTs were graded as having a high, unclear, or low risk of bias. Observational studies were evaluated according to the Newcastle–Ottawa scale (NOS). The NOS included 3 parts: patient selections, comparability of the study groups, and assessment of outcomes. Each part possessed a score of 4, 2, and 3. An overall quality score of ≥ 7 was defined as a high-quality study. If there were some disagreements, two other reviewers (Y.H1 and Y.H2) would be consulted.

### Quality of evidence

The overall certainty of evidence for each outcome was assessed using the Grading Recommendations Assessment, Development and Evaluation (GRADE) approach [21]. We used the Guideline Development Tool (https://www.gradepro.org) to formulate the Summary of Findings table.

### Statistical analysis

Statistical analysis was performed using RevMan version 5.4 (Cochrane Collaboration, London, UK). Using a random-effects model, the results were presented as risk ratio (RR) for dichotomous outcomes with the Mantel–Haenszel method and mean difference (MD) for continuous outcomes with the inverse variance method, all with 95% confidence intervals (CIs). The overall data were collected using a Z-test. All reported P values were two-sided, and a P-value < 0.05 was considered statistically significant. Statistical heterogeneity was estimated using the I^2^ statistic, which was considered significant above 50%. Subgroup analyses for primary outcome were performed for the following variables: (1) study design; (2) the presence of other agents; (3) the dose of IV iron; and (4) the time between iron therapy administration and surgery. Post hoc subgroup analysis was performed according to the control group. A funnel plot was used to estimate potential publication bias.

The results of a standard meta-analysis are often susceptible to type I or type II error due to repeated statistical testing or insufficient sample size [22]. To complement this meta-analysis, we performed a trial sequential analysis (TSA) to calculate the required heterogeneity-adjusted information size and trial sequential monitoring boundaries. The models for the transfusion rates were based on 0.05 for type 1 error, and 0.20 for type 2 error. TSA was performed in the TSA 0.9.5.10 Beta software.

## Results

### Identification and characteristics of eligible studies

**Fig. 1:**
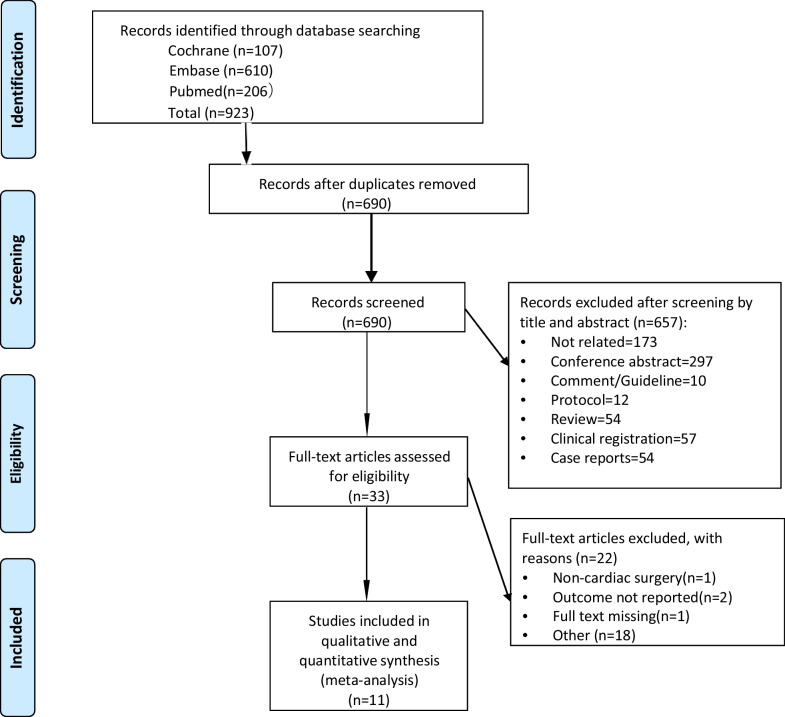
Study flowchart

The search yielded 923 citations (206 from PubMed, 610 from Embase and 107 from CENTRAL). We excluded 233 duplicates and a further 657 citations after title and abstract screening and assessed 33 full texts. Finally, 6 RCTs and 5 observational studies were included in this meta-analysis [3, 15–19, 23–27] (Fig. 1). The characteristics of the included studies are described in Table 1. There were 2286 patients included in this study. The sample size of the included studies ranged from 40 to 771 patients. Four RCTs compared IV iron (combined with other agents in three studies) with placebo [16, 24–26], 2 RCTs compared IV iron with oral iron [15, 23], and 5 observational studies compared IV iron with no treatment [3, 17–19, 27]. Additionally, although all studies investigated the effect of IV iron, the drug was administered at different dosages and time intervals. The dosage of IV iron ranged from 200 to 1000 mg per patient, and the time interval of IV iron administration ranged from one day before surgery to 10 weeks before surgery. Four RCTs and two observational studies noted elaborate transfusion triggers in their studies, with hemoglobin levels ranging from 7 to 8 g/L.

**Table 1:** Characteristics of included studies

Study	Design	Type of surgery	Inclusion criteria	Group (N)	IV iron time and dose	Other agents	Mean age (years)	Trigger for transfusion	NOS selection/comparability/outcome
Yoo 2011 (Korea)	RCT single-site single-blinded	Valve surgery	WHO anemia criteria	IV iron (37)	1 day before surgery 200 mg	500U/kg EPO	56 ± 12	Hb < 70 g/L during CPB or Hb < 80 g/L after CPB and postoperatively	/
				Control (37)	Saline		59 ± 12		
Urena 2017 (Canada)	RCT double-blind	Valve surgery or TAVI	WHO anemia criteria	IV iron (48)	10(± 4) and 1(± 1) days before surgery 200 mg each time	0.75 ug/kg EPO each time	81 ± 7	Hb ≤ 70 g/L or haematocrit less than 22%	/
				Control (52)	Saline		81 ± 7		
Padmanabhan 2019 (UK)	RCT single-centrenon-blinded	CABG or open valve surgery	WHO anemia criteria	IV iron (20)	3–8 weeks before surgery 1000 mg	None	73 ± 12	Not mentioned	/
				Control (20)	Oral iron		75 ± 10		
Spahn 2019 (Switzerland)	RCT single-centre double-blind	CABG and/or valve surgery	WHO anemia criteria or iron deficiency	IV iron (243)	The day before surgery 20 mg/kg (Max 1000 mg)	40,000U EPO + 1 mg VB_12_ + 5 mg FA	69 ± 11	Hb < 70-80 g/L intraoperatively and in intensive care or Hb < 80 g/L on the regular ward	/
				Control (241)	Placebo		67 ± 12		
Kong 2022 (UK)	RCT single-centre open-label	CABG and/or valve surgery	Hb: 100-130 g/L with iron deficiency	IV iron (79)	2–10 weeks before surgery 20 mg/kg (Max 1000 mg)	200ug EPO	74 ± 9	Hb ≤ 70 g/L	/
				Control (77)	Oral iron		73 ± 7		
Shokri 2022 (Egypt)	RCT double-blind	CABG	WHO anemia criteria	IV iron (40)	7 days before surgery 1000 mg	None	58.3 ± 4.4	Not mentioned	/
				Control (40)	Saline		60.1 ± 4.8		
Cladellas 2012 (Spain)	Prospective cohort study	Cardiac surgery	WHO anemia criteria	IV iron (75)	1 month before surgery Max 200 mg each time (five times in total)	500 IU/kg EPO each time	73 ± 10	Hb < 70 g/L	3/1/3
				Observation (59)	No treatment		71 ± 8		
Klein 2020 (UK)	Retrospective multicentre observational study	CABG and/or valve surgery	WHO anemia criteria	IV iron (64)	At least 10 days before surgery 20 mg/kg (Max1000mg)	None	70.2 ± 10.9	Not mentioned	4/1/3
				Observation (72)	No treatment		69.3 ± 11.8		
Evans 2021 (UK)	Retrospective observational study	CABG and/or valve surgery	Hb < 130 g/L	IV iron (75)	Before surgery 20 mg/kg	None	71 ± 11	Hb < 80 g/L	4/1/3
				Observation (72)	No treatment		72 ± 8		
Peel 2021 (Canada)	Retrospective cohort study	CABG and/or valve surgery	Hb < 130 g/L	IV iron (84)	Before surgery < 300 mg; 300-600 mg; > 600 mg	None	68 ± 13	Not mentioned	3/1/3
				Observation (78)	No treatment		68 ± 10		
Quarterman 2021 (UK)	Retrospective observational study	CABG and/or valve surgery	Hb < 130 g/L	IV iron (190)	2 weeks before surgery 20 mg/kg	None	70.30 ± 10.46	Not mentioned	4/1/3
				Observation (581)	No treatment		72.65 ± 8.17		

*RCT* randomized controlled trial, *IV* intravenous, *EPO* erythropoietin, *CABG* coronary artery bypass grafting, *VB*_12_ vitamin B_12_, *FA* folic acid

### Study quality

**Fig. 2:**
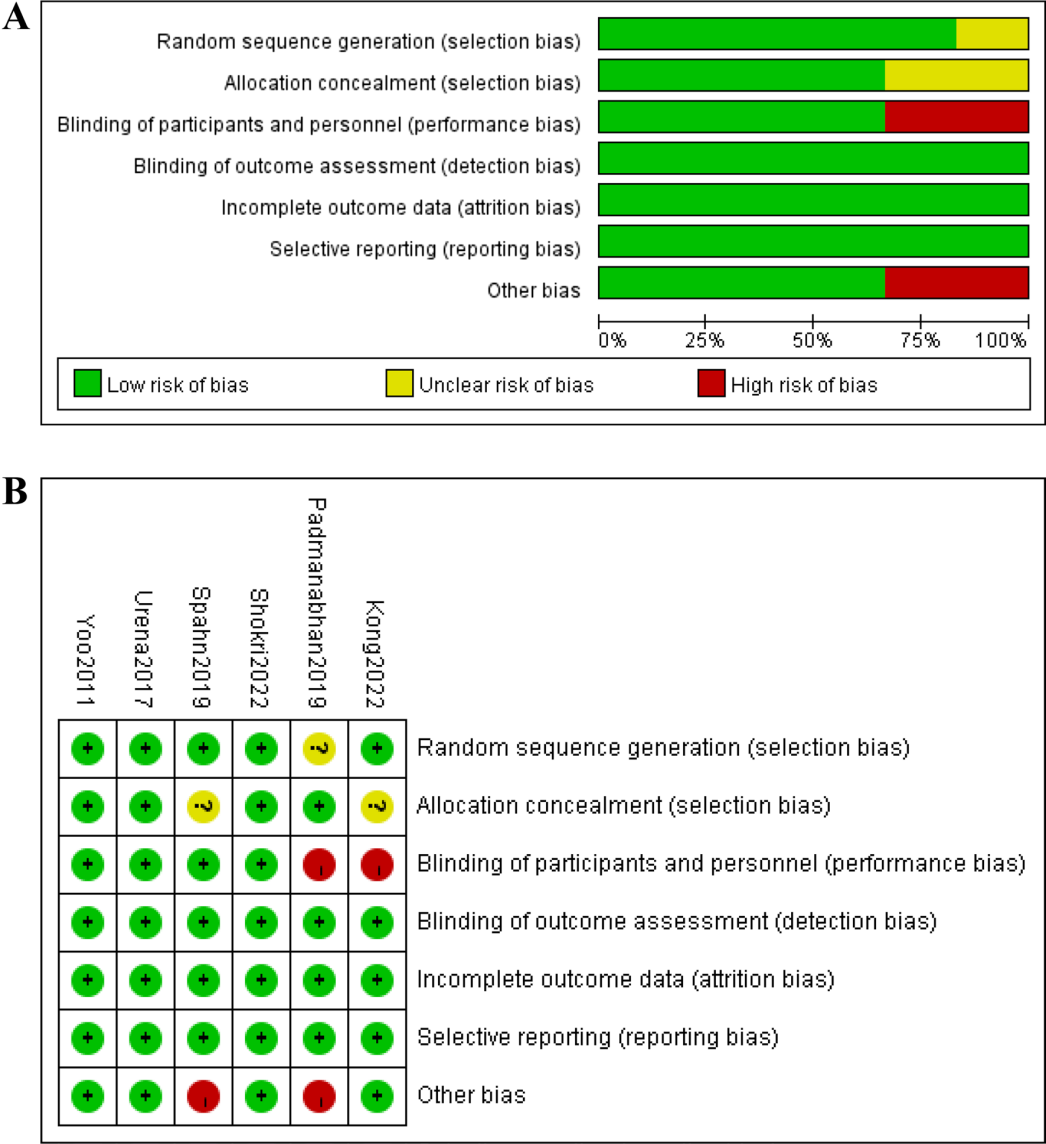
Methodological quality and bias risk. **a** Risk of bias graph for each included study; **b** Risk of bias summary. Green circle = low bias risk, red circle = high bias risk, yellow circle = unclear bias risk

The quality assessment of the included RCTs is shown in Fig. 2, and the quality assessment of observational studies is shown in Table 1. Study quality assessments showed that 3 of 6 RCTs [16, 25, 26] described the methods used for random sequence generation and allocation concealment and 4 of 6 RCTs [16, 24–26] conducted the blinding of participants and personnel. All studies were at low risk of bias in the blinding of outcome assessments due to the characteristics of the endpoint (transfusion rates). Quality appraisal of observational studies showed that all 5 studies were graded as high quality with a score of 7 or 8.

### Primary outcome: transfusion rate (%)

**Fig. 3:**
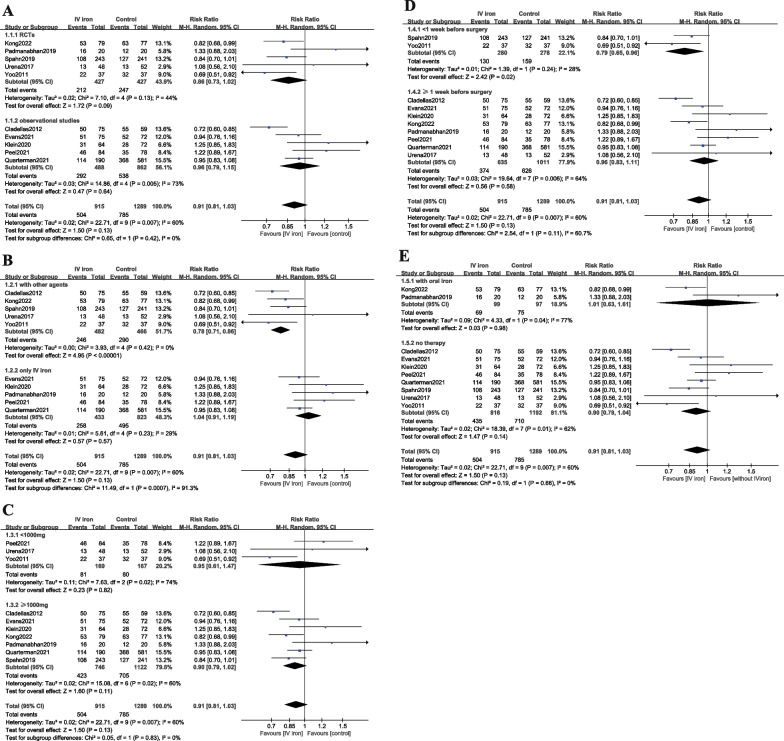
Forest plot comparing IV iron and control for transfusion rate. **a** Subgroup analysis according to study design; **b** Subgroup analysis according to the presence of other agents; **c** Subgroup analysis according to the dosage of IV iron; **d** Subgroup analysis according to the time interval between iron therapy administration and surgery;** e** Subgroup analysis according to the control group

There were 10 studies including 2204 patients reporting the proportion of patients who received erythrocyte transfusion intra- and postoperatively. Overall, the transfusion rate was 55.1% in patients receiving IV iron and 60.9% in patients not receiving IV iron. The RR (0.91, 95% CI 0.81–1.03, P = 0.13, P for heterogeneity = 0.007, I^2^ = 60%, low quality) (Fig. 3) did not reveal an association between IV iron therapy and a decreased transfusion rate. Subgroup analyses are also presented in Fig. 3.

### Secondary outcomes

#### Units transfused per patient

**Fig. 4:**
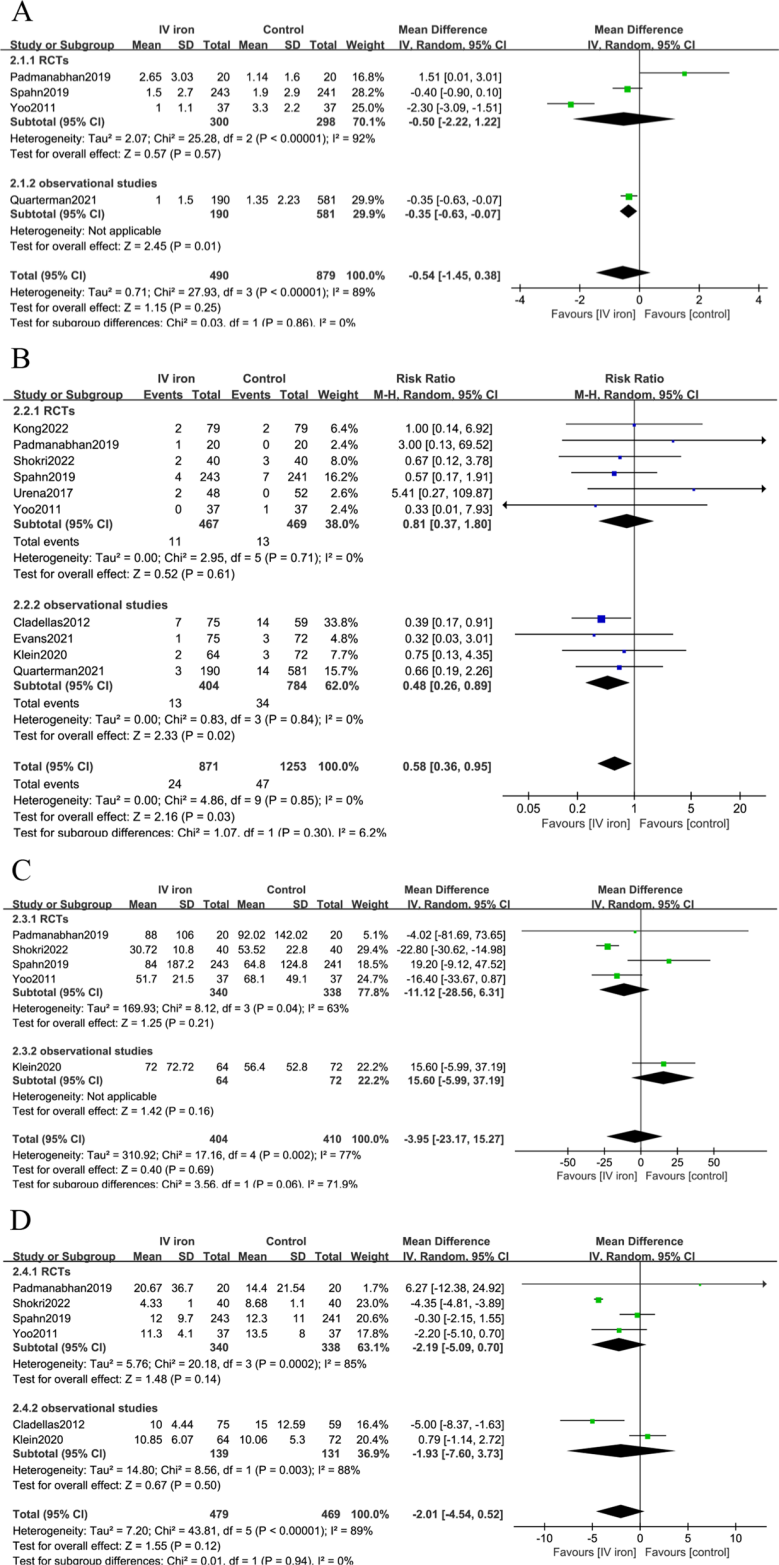
Forest plot comparing IV iron and control for secondary outcomes. **a** Forest plot for units transfused per patient; **b** Forest plot for mortality; **c** Forest plot for ICU stay; **d** Forest plot for hospital length of stay

There were 4 studies including 1369 patients reporting units transfused per patient. There was no significant difference in the units transfused per patient between the IV iron group and the control group during the intra- and postoperative periods (MD = − 0.54, 95% CI − 1.45 to 0.38, P = 0.25, P for heterogeneity < 0.001, I^2^ = 89%, very low quality) (Fig. 4a).

### Mortality

There were 10 studies including 2124 patients reporting all-cause mortality. The pooled analysis showed a significantly lower rate of mortality in the IV iron group than in the control group (2.76% vs 3.75%, RR = 0.58, 95% CI 0.36–0.95, P = 0.03, P for heterogeneity = 0.85, I^2^ = 0%, moderate quality). However, no mortality reduction existed when only RCTs were included (Fig. 4b).

### ICU stay

There were 5 studies including 814 patients reporting ICU stay (hours). There was no significant difference in the ICU stay between the IV iron group and the control group (MD = − 3.95, 95% CI − 23.17 to 15.27, P = 0.69, P for heterogeneity = 0.002, I^2^ = 77%, very low quality) (Fig. 4c).

### Hospital LOS

There were 6 studies including 948 patients reporting hospital LOS. There was no significant difference in hospital LOS between the IV iron group and the control group (MD = − 2.01, 95% CI − 4.54 to 0.52, P = 0.12, P for heterogeneity < 0.001, I^2^ = 89%, very low quality) (Fig. 4d).

### Adverse events

Pooled data showed that there were no significant differences with the use of IV iron compared with the control group in renal adverse events (14.5% vs 18.8%, RR = 0.72, 95% CI 0.50–1.05, P = 0.09, P for heterogeneity = 0.16, I^2^ = 37%) from 6 studies; cardiac adverse events (15.1% vs 18.9%, RR = 0.81, 95% CI 0.62–1.07, P = 0.13, P for heterogeneity = 0.88, I^2^ = 0%) from 6 studies; and cerebral adverse events (3.3% vs 1.9%, RR = 1.62, 95% CI 0.85–3.10, P = 0.15, P for heterogeneity = 0.47, I^2^ = 0%) from 5 studies (Additional file 1 : Fig. 1).

### Sensitivity analysis

Subgroup analyses were conducted for primary outcome according to study design (RCTs or observational studies), the presence of other agents (with other agents or only IV iron), the dose of IV iron (< 1000 mg or ≥ 1000 mg), and the time interval between iron therapy administration and surgery (< 1 week or ≥ 1 week) and control group (with oral iron or no therapy) (Fig. 3). The pooled data of the transfusion rate from RCTs or observational studies showed no change. The analyses showed that the application of other agents (mainly EPO) and IV iron therapy < 1 week before surgery were beneficial for decreasing transfusion rates. In addition, there was no significant difference in the transfusion rate between the two different dosages of IV iron. In addition, post hoc subgroup analysis by control group showed that the control group did not have an impact on the transfusion rate.

### Publication bias

We evaluated publication bias by the Funnel plot. Funnel plots for transfusion rate and mortality were relative symmetry (Additional file 1 : Fig. 2).

### TSA

**Fig. 5:**
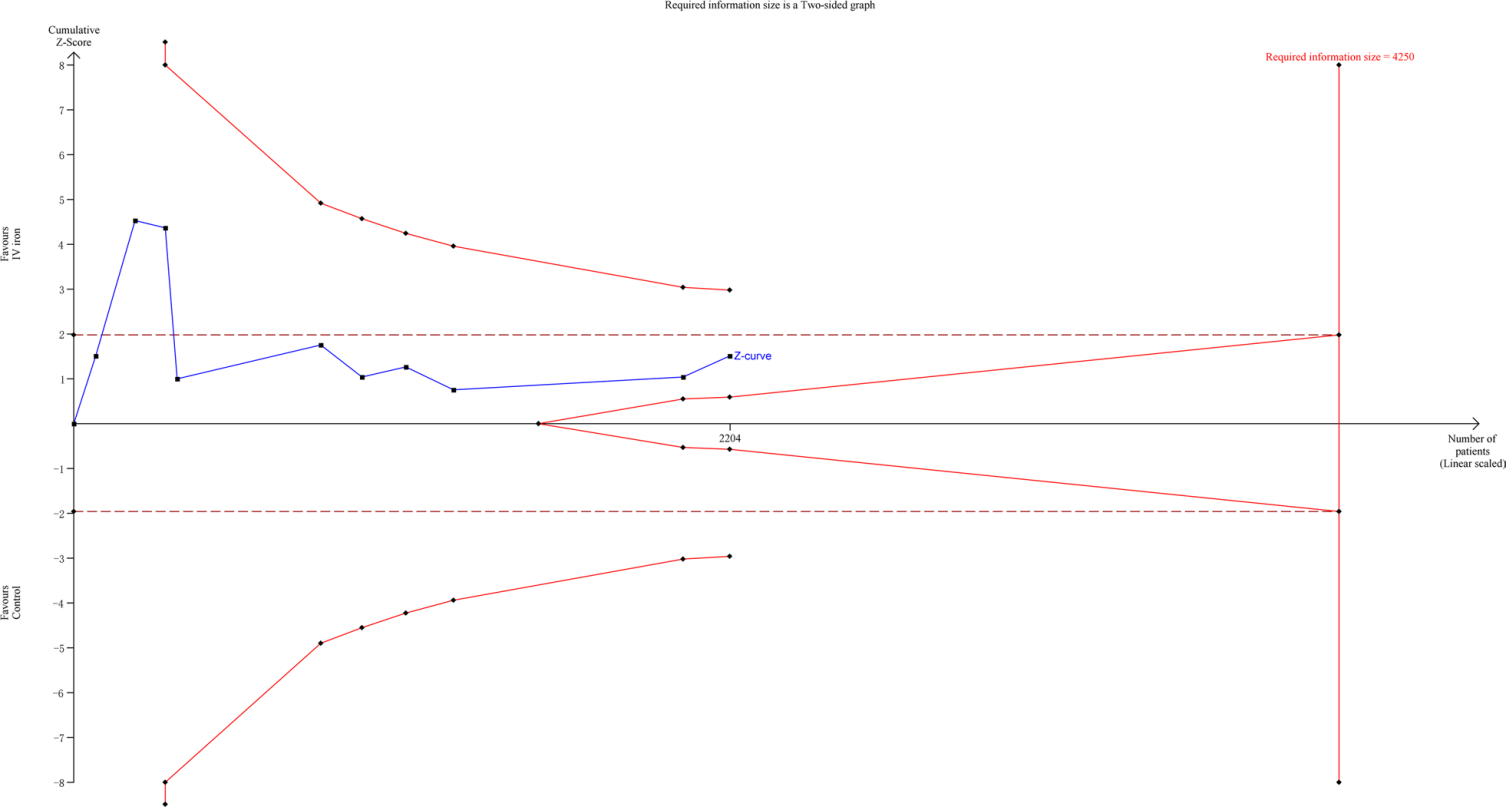
TSA for transfusion rate. RIS (required information size = 4250)

The TSA of the transfusion rate is presented in Fig. 5. The required heterogeneity-adjusted information size was 4250. The cumulative Z curve neither crossed the traditional significance boundary nor reached the required information size, which indicated that more trials were needed to reliably detect the effect of IV iron on the transfusion rate in cardiac surgery patients with anemia.

### Strength of evidence

GRADE system grades of evidence are low certainty for transfusion rate, very low certainty for units transfused per patient, ICU stay, hospital LOS and adverse events, moderate certainty for mortality. The results are summarized in Additional file 1: C.

## Discussion

The main finding of this meta-analysis showed that IV iron for the treatment of anemia before cardiac surgery was not associated with a reduced transfusion rate when compared with the control group. In addition, IV iron decreased the incidence of all-cause mortality. However, units transfused per patient, ICU stay, hospital LOS and adverse events did not differ between the IV iron group and the control group.

### Comparison with previous meta-analyses

There was actually one review [14] and one meta-analysis [13] reporting intravenous (IV) iron therapy for patients undergoing cardiac surgery. And these two studies were different in both population and outcomes from this meta-analysis. First, 2 of 6 studies included non-anemic participants in Tankard et al.’s study [14] and 6 of 7 observational studies had no clear participants inclusion criteria in Gupta et al.’s study [13]. Second, the primary outcome was different. Tankard et al. just overviewed the findings of included studies in transfusion rate without concrete data processing and Gupta et al. set transfusion rate as a secondary outcome.

Because more than half of anemic patients undergoing cardiac surgery have iron deficiency, IV iron therapy preoperatively is recommended [2, 28, 29]. However, the evidence regarding the effect of IV iron on reducing the transfusion rate for cardiac surgery patients with preoperative anemia is limited and unclear [2, 13, 14, 30].This systematic review and meta-analysis suggested that IV iron did not decrease transfusion rates compared with the control group, which was not consistent with Gupta et al. [13] or Elhenawy et al. [31] as they found a significant association of IV iron treatment with a reduced transfusion rate in cardiac surgery patients (RR 0.81, 95% CI 0.70–0.94, P = 0.005) or other major surgery patients (RR 0.84, 95% CI 0.70 to 0.99, P = 0.04). However, their meta-analysis [13, 31] included studies exploring the efficacy of IV iron in nonanemic patients. Nonanemic patients were less likely to receive blood transfusion than anemic patients [32, 33], resulting in overall transfusion rates in these studies [13, 31] decreasing. However, this positive change may be caused by participants’ characteristics or IV iron therapy, and thus the benefits of IV iron on reducing the transfusion requirement for patients with anemia may be obfuscated. In addition, the application of TSA indicated that the merits of IV iron therapy in anemic cardiac surgery patients require further trials.

Furthermore, subgroup analyses (Fig. 3) for the primary outcome were performed. The subgroup analyses by the presence of other agents and by the time interval between iron therapy administration and surgery suggested that the application of other agents (mainly EPO) and IV iron therapy < 1 week before surgery were beneficial for decreasing transfusion rates. Although the dosage of EPO varied in five studies and influenced the externality of this practice, some meta-analyses [34, 35] suggested that preoperative iron (enteral or IV) plus EPO therapy decreased the need for erythrocyte transfusion in anemic surgical patients, which was concordant with our study. The results of subgroup analysis by the time interval of IV iron administration were contradictory to those of other studies [36, 37], probably due to limited studies and sample sizes. However, from a practical perspective, initiating IV iron therapy the week prior to surgery is also suggested [38]. Taken together, future studies with a sufficiently large sample size are needed to focus on patients undergoing cardiac surgery. Further data are also required to examine the role of IV iron in three aspects: [1] applying IV iron with or without other agents (mainly EPO); [2] dosage of IV iron; and [3] time of IV iron administration preoperatively [33].

Meanwhile, IV iron was associated with a reduction in mortality, which was concordant with Gupta et al. [13]. In addition, there were no significant differences in units transfused per patient, ICU stay, hospital LOS or adverse events between the two groups. These secondary outcomes were consistent with other meta-analyses [31, 39, 40]. Nonetheless, we cannot conclude that there was no association between IV iron and these secondary outcomes in cardiac surgery. The different follow-up times and limited trials exploring these secondary outcomes potentially introduced heterogeneity.

Therefore, the ongoing trials, the Evaluating the Efficacy of Erythropoietin and Intravenous Iron on Transfusion Requirements in Patients Undergoing Cardiac Surgery (IRCT20190121042447N1) and the Intravenous Iron for Treatment of Anemia Before Cardiac Surgery (NCT02632760) [1] are anticipated to elucidate the impact of preoperative IV iron for anemic patients undergoing cardiac surgery.

Several limitations should be considered in our study. First, the conclusion on the internal and external validity of the finding of primary outcome was drawn with caution due to a limited high-quality RCTs and sample size. Second, the certainty of our findings ranked very low to moderate because of the inclusion of trials with a high risk of bias and observational studies. Third, the dosage of IV iron and the time of IV iron administration varied among studies. Although we performed subgroup analyses to control some confounding factors, it is difficult to obtain high-quality conclusions from the pooled studies. Fourth, transfusion triggers were not confirmed in the included studies, which may have influenced the transfusion rate. However, we deemed that patient blood management in diverse hospitals was similar and guided by the international guidelines. In addition, we did not extract data that might help elucidate a potential cause for the decreased mortality, given that were no differences in the other outcomes measured. Finally, the result that IV iron therapy was not associated with reduced transfusion rates is not yet conclusive and requires further trials to affirm as indicated in TSA.

## Conclusion

IV iron treatment for patients with anemia before cardiac surgery did not reduce the transfusion requirement (low quality), but it was associated with decreased mortality (moderate quality). Further large-scale, high-quality randomized clinical trials are warranted to confirm or refute our findings.

## Supplementary Information

**Additional file 1.** Supplementary file.

## Data Availability

All data generated or analyzed during this study were included in this published article.

## Abbreviations

ABT: Allogeneic blood transfusion
CI: Confidence interval
EPO: Erythropoietin
ICU: Intensive care unit
IV: Intravenous
LOS: Length of hospital stay
MD: Mean difference
RCTS: Randomized controlled trials
RR: Risk ratio
TSA: Trial sequential analysis

## References

[1] Myles PS, Richards T, Klein A, Smith J, Wood EM, Heritier S (2021). Rationale and design of the intravenous iron for treatment of anemia before cardiac surgery trial. Am Heart J.

[2] Yang SS, Al Kharusi L, Gosselin A, Chirico A, Baradari PG, Cameron MJ (2022). Iron supplementation for patients undergoing cardiac surgery: a systematic review and meta-analysis of randomized controlled trials. Can J Anaesthesia.

[3] Klein AA, Chau M, Yeates JA, Collier T, Evans C, Agarwal S (2020). Preoperative intravenous iron before cardiac surgery: a prospective multicentre feasibility study. Br J Anaesth.

[4] Chandra S, Kulkarni H, Westphal M (2017). The bloody mess of red blood cell transfusion. Critical Care (London, England).

[5] Mueller MM, Van Remoortel H, Meybohm P, Aranko K, Aubron C, Burger R (2019). Patient blood management: recommendations from the 2018 Frankfurt consensus conference. JAMA.

[6] Musallam KM, Tamim HM, Richards T, Spahn DR, Rosendaal FR, Habbal A (2011). Preoperative anaemia and postoperative outcomes in non-cardiac surgery: a retrospective cohort study. Lancet.

[7] Meybohm P, Westphal S, Ravn HB, Ranucci M, Agarwal S, Choorapoikayil S (2020). Perioperative anemia management as part of PBM in cardiac surgery: a narrative updated review. J Cardiothorac Vasc Anesth.

[8] DeLoughery TG (2014). Microcytic anemia. N Engl J Med.

[9] Kassebaum NJ (2016). The global burden of Anemia. Hematol Oncol Clin North Am.

[10] de Las-Nieves-Lopez MA, Matas Cobos AM, Sarria Gonzalez F, Dominguez Lomeña MJ, Palomo Hernandez AM, Gil Gines E (2018). Red blood cell transfusion after a global strategy for early detection and treatment of iron deficiency anemia: three-year results of a prospective observational study. Transfusion.

[11] Padhi S, Kemmis-Betty S, Rajesh S, Hill J, Murphy MF (2015). Blood transfusion: summary of NICE guidance. BMJ.

[12] Muñoz M, Acheson AG, Auerbach M, Besser M, Habler O, Kehlet H (2017). International consensus statement on the peri-operative management of anaemia and iron deficiency. Anaesthesia.

[13] Gupta S, Panchal P, Gilotra K, Wilfred AM, Hou W, Siegal D (2020). Intravenous iron therapy for patients with preoperative iron deficiency or anaemia undergoing cardiac surgery reduces blood transfusions: a systematic review and meta-analysis. Interact Cardiovasc Thorac Surg.

[14] Tankard KA, Park B, Brovman EY, Bader AM, Urman RD (2020). The impact of preoperative intravenous iron therapy on perioperative outcomes in cardiac surgery: a systematic review. J Hematol.

[15] Kong R, Hutchinson N, Hill A, Ingoldby F, Skipper N, Jones C (2022). Randomised open-label trial comparing intravenous iron and an erythropoiesis-stimulating agent versus oral iron to treat preoperative anaemia in cardiac surgery (INITIATE trial). Br J Anaesth.

[16] Shokri H, Ali I (2022). Intravenous iron supplementation treats anemia and reduces blood transfusion requirements in patients undergoing coronary artery bypass grafting—a prospective randomized trial. Ann Card Anaesth.

[17] Evans CR, Jones R, Phillips G, Greene G, Phillips M, Morris-Clarke R (2021). Observational study of pre-operative intravenous iron given to anaemic patients before elective cardiac surgery. Anaesthesia.

[18] Peel JK, Trudeau J, Tano R, Jadunandan S, Callum J, Moussa F (2021). Determining optimal treatment to correct preoperative anemia and reduce perioperative allogeneic blood transfusions in cardiac surgery: a retrospective cohort study. J Cardiothorac Vasc Anesth.

[19] Quarterman C, Shaw M, Hughes S, Wallace V, Agarwal S (2021). Anaemia in cardiac surgery—a retrospective review of a centre's experience with a pre-operative intravenous iron clinic. Anaesthesia.

[20] Liberati A, Altman DG, Tetzlaff J, Mulrow C, Gøtzsche PC, Ioannidis JP (2009). The PRISMA statement for reporting systematic reviews and meta-analyses of studies that evaluate health care interventions: explanation and elaboration. Ann Intern Med.

[21] Guyatt GH, Oxman AD, Vist GE, Kunz R, Falck-Ytter Y, Alonso-Coello P (2008). GRADE: an emerging consensus on rating quality of evidence and strength of recommendations. BMJ (Clinical research ed).

[22] Claire R, Gluud C, Berlin I, Coleman T, Leonardi-Bee J (2020). Using Trial Sequential Analysis for estimating the sample sizes of further trials: example using smoking cessation intervention. BMC Med Res Methodol.

[23] Padmanabhan H, Siau K, Nevill AM, Morgan I, Cotton J, Ng A (2019). Intravenous iron does not effectively correct preoperative anaemia in cardiac surgery: a pilot randomized controlled trial. Interact Cardiovasc Thorac Surg.

[24] Spahn DR, Schoenrath F, Spahn GH, Seifert B, Stein P, Theusinger OM (2019). Effect of ultra-short-term treatment of patients with iron deficiency or anaemia undergoing cardiac surgery: a prospective randomised trial. Lancet (london, england).

[25] Urena M, Del Trigo M, Altisent OA, Campelo-Prada F, Regueiro A, DeLarochellière R (2017). Combined erythropoietin and iron therapy for anaemic patients undergoing transcatheter aortic valve implantation: the EPICURE randomised clinical trial. EuroIntervention.

[26] Yoo YC, Shim JK, Kim JC, Jo YY, Lee JH, Kwak YL (2011). Effect of single recombinant human erythropoietin injection on transfusion requirements in preoperatively anemic patients undergoing valvular heart surgery. Anesthesiology.

[27] Cladellas M, Farré N, Comín-Colet J, Gómez M, Meroño O, Bosch MA (2012). Effects of preoperative intravenous erythropoietin plus iron on outcome in anemic patients after cardiac valve replacement. Am J Cardiol.

[28] Auerbach M, Gafter-Gvili A, Macdougall IC (2020). Intravenous iron: a framework for changing the management of iron deficiency. Lancet Haematol.

[29] Corwin HL, Shander A, Speiss B, Muñoz M, Faraoni D, Calcaterra D (2022). Management of perioperative iron deficiency in cardiac surgery: a modified RAND Delphi Study. Ann Thorac Surg.

[30] Spahn DR, Kaserer A (2022). Intravenous iron for all in cardiac surgery?. Ann Surg.

[31] Elhenawy AM, Meyer SR, Bagshaw SM, MacArthur RG, Carroll LJ (2021). Role of preoperative intravenous iron therapy to correct Anemia before major surgery: a systematic review and meta-analysis. Syst Rev.

[32] Pothof AB, Bodewes TCF, O'Donnell TFX, Deery SE, Shean K, Soden PA (2018). Preoperative Anemia is associated with mortality after carotid endarterectomy in symptomatic patients. J Vasc Surg.

[33] Quarterman C, Agarwal S (2021). Pre-operative optimisation with intravenous iron in cardiac surgery: a reply. Anaesthesia.

[34] Kaufner L, von Heymann C, Henkelmann A, Pace NL, Weibel S, Kranke P (2020). Erythropoietin plus iron versus control treatment including placebo or iron for preoperative anaemic adults undergoing non-cardiac surgery. Cochrane Database Systemat Rev.

[35] Kei T, Mistry N, Curley G, Pavenski K, Shehata N, Tanzini RM (2019). Efficacy and safety of erythropoietin and iron therapy to reduce red blood cell transfusion in surgical patients: a systematic review and meta-analysis. Canad J Anaesthesia..

[36] Neef V, Baumgarten P, Noone S, Piekarski F, Triphaus C, Kleinerüschkamp A (2021). The impact of timing of intravenous iron supplementation on preoperative haemoglobin in patients scheduled for major surgery. Blood Transf.

[37] Triphaus C, Judd L, Glaser P, Goehring MH, Schmitt E, Westphal S (2021). Effectiveness of preoperative iron supplementation in major surgical patients with iron deficiency: a prospective observational study. Ann Surg.

[38] Rubinger DA, Cahill C, Ngo A, Gloff M, Refaai MA (2020). Preoperative Anemia management: What’s new in 2020?. Curr Anesthesiol Rep.

[39] Ng O, Keeler BD, Mishra A, Simpson JA, Neal K, Al-Hassi HO (2019). Iron therapy for preoperative Anaemia. Cochrane Database System Rev..

[40] Shah A, Palmer AJR, Fisher SA, Rahman SM, Brunskill S, Doree C (2018). What is the effect of perioperative intravenous iron therapy in patients undergoing non-elective surgery? A systematic review with meta-analysis and trial sequential analysis. Perioperat Med.

